# The value of Gd-EOB-DTPA-enhanced MR imaging in characterizing cirrhotic nodules with atypical enhancement on Gd-DTPA-enhanced MR images

**DOI:** 10.1371/journal.pone.0174594

**Published:** 2017-03-29

**Authors:** Yi-Chun Wang, Chen-Te Chou, Ching-Po Lin, Yao-Li Chen, Yung-Fang Chen, Ran-Chou Chen

**Affiliations:** 1 Department of Biomedical Imaging and Radiological Sciences, National Yang-Ming University, Taipei City, Taiwan; 2 Department of Radiology, China Medical University Hospital, Taichung City, Taiwan; 3 Department of Radiology, Changhua Christian Hospital, Changhua City, Taiwan; 4 School of Medicine, Kaohsiung Medical University, Kaohsiung City, Taiwan; 5 Department of Surgery, Changhua Christian Hospital, Changhua City, Taiwan; 6 Health Promotion Administration, Ministry of Health and Welfare, Taipei City, Taiwan; Chang Gung Memorial Hospital Kaohsiung Branch, TAIWAN

## Abstract

**Purpose:**

To evaluate the utility of Gd-EOB-DTPA-enhanced magnetic resonance imaging (MRI) in characterizing atypically enhanced cirrhotic nodules detected on conventional Gd-DTPA-enhanced MR images.

**Materials and methods:**

We enrolled 61 consecutive patients with 88 atypical nodules seen on conventional Gd-DTPA-enhanced MR images who underwent Gd-EOB-DTPA-enhanced MRI within a 3-month period. Using a reference standard, we determined that 58 of the nodules were hepatocellular carcinoma (HCC) and 30 were dysplastic nodules (DNs). Tumor size, signal intensity on precontrast T1-weighted images (T1WI), T2-weighted images (T2WI) and diffusion-weighted images (DWI), and the enhancement patterns seen on dynamic phase and hepatocyte phase images were determined.

**Results:**

There were significant differences between DNs and HCC in hyperintensity on T2WI, hypointensity on T1WI, hypervascularity on arterial phase images, typical HCC enhancement patterns on dynamic MR images, hypointensity on hepatocyte phase images, and hyperintensity on DWI. The sensitivity and specificity were 79.3% and 83.3% for T2WI, 50.0% and 80.0% for T1WI, 82.8% and 76.7% for DWI, 17.2% and 100% for dynamic MR imaging, 93.1% and 83.3% for hepatocyte phase imaging, and 46.8% and 100% when arterial hypervascularity was combined with hypointensity on hepatocyte-phase imaging.

**Conclusion:**

Gd-EOB-DTPA-enhanced hepatocyte phase imaging is recommended for patients at high risk for HCC who present with atypical lesions on conventional Gd-DTPA-enhanced MR images.

## Introduction

Hepatocellular carcinoma (HCC) is one of the leading causes of cancer-related death and patients with cirrhosis are at the highest risk of developing the disease. [1] According to guidelines published by the American Association for the Study of Liver Diseases (AASLD), a diagnosis of HCC can be established when nodules greater than 1.0 cm show arterial phase hypervascularity and venous/late phase contrast washout on dynamic computed tomographic (CT) or magnetic resonance (MR) images. [2] Small HCCs, however, are challenging to diagnose as they frequently present with atypical enhancement patterns in dynamic studies. In addition, it is important to differentiate benign cirrhotic nodules from atypical HCC because the treatment strategy for atypical HCC differs markedly from that for benign cirrhotic nodules. [3]

Gadoxetic acid (Gd-EOB-DTPA) is a liver-specific MR imaging (MRI) contrast medium that combines the properties of a conventional extracellular fluid contrast agent, thus enabling dynamic perfusion imaging, with that of a selective hepatocyte uptake agent, which allows for the evaluation of biliary excretion and delayed hepatobiliary imaging. This contrast agent has been demonstrated to increase the detection of focal liver lesions and to provide differential diagnostic information comparable to nonspecific extracellular gadolinium chelates. [4–9] Although the current guidelines of the Japan Society of Hepatology (JSH) recommend the use of gadoxetic acid-enhanced MRI to differentiate among hepatic lesions, [9] it remains to be determined whether this contrast agent is also effective at characterizing HCCs with atypical features seen on conventional gadolinium (Gd-DTPA)-enhanced MR images. The purpose of this study was to evaluate the utility of Gd-EOB-DTPA-enhanced MRI in characterizing atypically enhanced cirrhotic nodules detected on conventional Gd-DTPA-enhanced MR images.

## Materials and methods

### Patients

**Fig 1:**
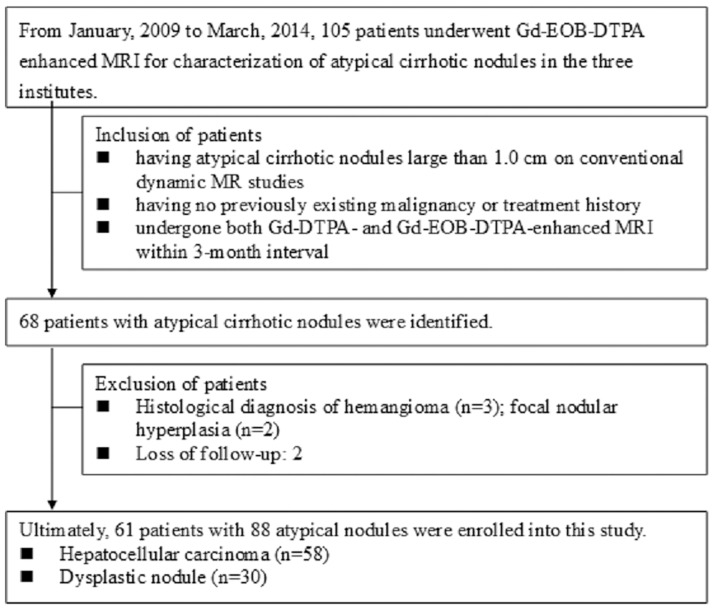
Flowchart showing patient selection.

This retrospective study was approved by the institutional review board of Taipei City Hospital (IRB: TCHIRB-1031009-E). Patients at risk for HCC were included in the study if they had evidence of atypical cirrhotic nodules measuring ≥ 1.0 cm on conventional Gd-DTPA-enhanced dynamic studies, had no history of malignancy and had undergone both Gd-DTPA- and Gd-EOB-DTPA-enhanced MRI within a 3-month interval during the period January 2009 to March 2014. A total of 61 patients (45 men and 16 women; mean age, 60 years; range, 50–70 years) fulfilled the inclusion criteria and were enrolled from three institutes (Fig 1). All patients had histologic evidence of liver cirrhosis including hepatitis B virus-related cirrhosis (n = 40), hepatitis C virus-related cirrhosis (n = 10), alcohol-induced cirrhosis (n = 3), combined hepatitis B and C virus-related cirrhosis (n = 1), and cryptogenic cirrhosis (n = 7). In patients with multiple atypical cirrhotic nodules (more than 3 nodules), only the largest three tumors were chosen for evaluation. The clinical characteristics of the study patients are presented in Table 1.

**Table 1:** Clinical characteristics of the 61 patients with atypically enhanced cirrhotic nodules on conventional dynamic MR images.

		Total number of patients N = 61
Age (year)		60.2 ±10.1
Gender	Male	45
	Female	16
Underlying disease	HBV	40
	HCV	10
	HBV+HCV	1
	Alcoholic	3
	Cryptogenic	7
Child-Pugh class	A	50
	B	9
	C	2
Mean size (cm)	HCC (n = 58)	2.1 ± 0.9
	DN (n = 30)	1.8 ± 0.5
AFP	normal < 20 ng/ml	37
	abnormal ≥ 20 ng/ml	24
AST(U/L)		52.7 ± 30.9
ALT(U/L)		43.3 ± 30.0

AASLD, American Association for the Study of Liver Diseases; HCC, hepatocellular carcinoma; SD, standard deviation; HBV, hepatitis B virus; HCV, hepatitis C virus; DN, dysplastic nodule; AFP, alfa-fetal protein; AST, aspartate aminotransferase; ALT, alanine aminotransferase. Values depicted as mean ± SD.

Among the 61 patients, six patients (9.8%) had more than three atypical cirrhotic nodules, 15 patients (24.6%) had two atypical nodules, and 40 patients (65.6%) had one atypical nodule detected on conventional Gd-DTPA-enhanced MR images. In total, 88 atypical cirrhotic nodules were chosen for analysis. The diagnosis of HCC was established solely on the basis of histologic examination of biopsy specimens. The diagnosis of a dysplastic nodule (DN) was based on the results of histologic examination of biopsy specimens or on the presence of changes in size or enhancement pattern of nodules during long-term follow-up.

Characterization of the vast majority (77.3%, 68/88) of nodules was based on histopathologic analysis of core needle biopsy specimens taken from 8 patients (11.8%, 8/68) and surgical specimens taken from 31 patients (88.2%, 60/68). The remaining 20 nodules (22.7%, 20/88) were diagnosed as DNs based not on histologic examination but on the fact that they did not show changes in size or enhancement pattern on CT or MR images that had been taken at three-month intervals over a two-year follow-up period. Using a reference standard, we identified 30 of the 88 nodules as dysplastic nodules (mean size, 1.8±0.5 cm; range, 0.9–2.6 cm) and the remaining 58 nodules as HCC (mean size, 2.1±0.9cm; range, 1.0–4.6 cm).

### MR imaging

The MR imaging protocol in this study was similar to that described in our previous study. [10] Briefly, MR imaging of the liver was performed with a 1.5 T MRI scanner (Achieva, Philips Healthcare, Eindhoven, The Netherlands; Magnetom Avanto, Siemens Healthcare, Erlangen, Germany; and Signa HDx, GE Healthcare, Milwaukee, WI, USA) equipped with a phased-array body coil. Prior to administration of contrast material, axial breath-holding dual echo T1-weighted (T1W) imaging was performed. Diffusion-weighted imaging (DWI) was obtained with a single-shot, spin-echo echo-planar imaging sequence using a parallel technique in the axial plane. The motion-probing gradients were applied sequentially in three orthogonal directions (x, y and z) to generate diffusion-weighted images.

All patients received 0.1 mmol/kg Gd-DTPA (Magnevist; Bayer Schering Pharma AG, Berlin, Germany) for gadolinium-enhanced MRI and 0.025 mmol/kg Gd-EOB-DTPA (Primovist; Bayer Schering Pharma AG) for gadoxetic-enhanced MRI. A bolus injection of contrast medium was administered at a speed of 2 mL/second through a peripheral vein and then the line was flushed with normal saline (20 mL). A care bolus technique was used to establish the optimal timing for the arterial phase. Dynamic three-dimensional T1-weighted images were acquired before injection of contrast agent and then in four phases after administration of Gd-DTPA, namely the arterial phase (AP, about 15–20 seconds), the portal venous phase (PP, about 50–56 seconds), the venous phase (VP, about 85–95 seconds), and the delayed phase (DP,180 seconds). Additional hepatocyte-phase (HP) images were acquired 20 minutes after the injection of Gd-EOB-DTPA. The parameters used to obtain the pulse sequences at the three hospitals are shown in Table 2. An integrated parallel acquisition technique (acceleration factor of 2) and a motion correction technique were applied to improve image quality.

**Table 2:** The parameters of MR pulse sequences used in the three institutes.

		T2WI	T1WI	Dynamic study	DWI
H1	Sequence	TSE	GE	3D T1 VIBE	SS-SE EPI
	Matrix	320 x 320	192 x 256	192 x 256	192 x 256
	TE(ms)	84	2.3/4.4	1.4–1.6	86
	TR(ms)	3600–4000	140	3.5–3.7	5800
	Flip angle	140°	70°	70°	90°
	Slice thickness	5 mm	5 mm	3 mm	7 mm
	b value				0, 100, 500, 1000
H2	Sequence	TSE	GE	3D T1 THRIVE	SS-SE EPI
	Matrix	192 x 256	192 x 256	192 x 256	192 x 256
	TE(ms)	110	2.3/4.6	3.3	70
	TR(ms)	1000	180	6.7	1500
	Flip angle	90°	10°	10°	90°
	Slice thickness	8 mm	8 mm	5 mm	7 mm
	b value				0, 100, 500, 1000
H3	Sequence	FSE	GE	3D T1 LAVA	SS-SE EPI
	Matrix	352 x 224	288 x 192	288 x 192	128 x 128
	TE(ms)	10.5–91.3	4.2/1.8	2–11	57.5
	TR(ms)	3–15000	105~130	4.1	2000
	Flip angle	90°	70°	12°	90°
	Slice thickness	6 mm	6 mm	6 mm	6 mm
	b value				0, 500

H1, hospital 1; H2, hospital 2; H3 hospital 3; T2WI, T2-weighted imaging; T1WI, T1-weighted imaging; DWI, diffusion-weighted imaging; TSE, turbo spin echo; GE, gradient echo; 3D, three dimension; VIBE, volumetric interpolated breath-hold examination; SS-SE, single shot spin echo, EPI, echo planar imaging; THRIVE, high resolution isotropic volume examination; FSE, fast spin echo; LAVA, liver acquisition with volume acceleration

### Imaging evaluation

Images were evaluated as described in our previous study. [10] Briefly, images were evaluated on a dual-screen diagnostic workstation (GE Healthcare, Milwaukee, WI, USA). For the assessment of each image, liver maps were created by one of the authors by drawing each individual liver lesion on a respective map, according to the Couinaud system of liver anatomy. All imaging results were independently analyzed using visual assessment by two radiologists with more than 10 years of experience in abdominal MR imaging. The two observers were blinded to the clinical information and final diagnosis, and they recorded the signal intensity on pre-contrast T1WI/T2WI/DWI, the enhancement patterns on dynamic MR images, and signal intensity on hepatocyte-phase images. Signal intensity of the focal liver nodules on dual echo T1WI, T2WI, DWI, and hepatocyte-phase images was classified as hypointense, isointense, or hyperintense relative to the adjacent liver parenchyma. Arterial enhancement was detected by automatic subtraction using the software provided by the MR manufacturer. If a marked subtraction artifact due to position change was found, then manual evaluation of the changes in signal intensity of the lesion relative to those seen in the surrounding liver parenchyma for each phase of the dynamic study was done using an operator-defined region of interest (ROI). A circular ROI was drawn to encompass as much of the lesion as possible. The enhancement pattern of the HCC (relative to the adjacent liver parenchyma) was classified as hypovascular, isovascular, or hypervascular enhancement. Any difference of opinion between the two reviewers was resolved by a third radiologist who was also blinded to the clinical information and final diagnosis.

### Statistical analysis

The Fisher’s exact test was used to compare categorical variables, such as enhancement pattern on dynamic studies and signal intensity on MR images. Continuous variables, such as age and tumor size, were analyzed by the independent-*t* test. Sensitivities, specificities, positive and negative predictive values, and positive and negative likelihood ratios of all paired MRI features were evaluated by calculating the areas under the receiver operating characteristic (ROC) curves (Az). Diagnostic accuracies between all paired MRI features were also compared using MedCalc software (version 13.1.2; MedCalc, Mariakerke, Belgium). The optimal cutoff values were chosen by maximizing the Youden index on the estimated curves (sensitivity + specificity-1). A *p*-value of less than 0.05 was considered to indicate statistical significance. All statistical analyses were performed with the statistical package SPSS (Version 14.0, SPSS, Chicago, IL).

## Results

**Fig 2:**
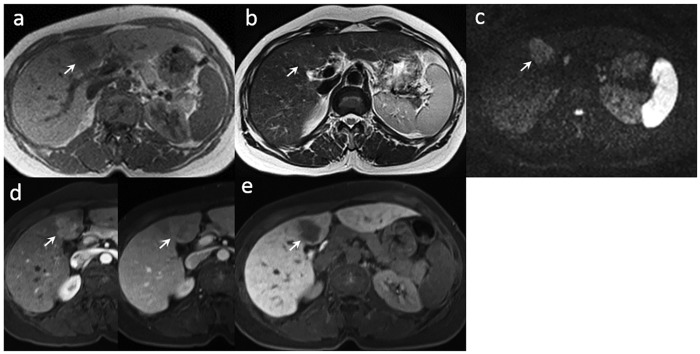
A 50-year-old female with moderately differentiated HCC (arrow) at S4 of the liver underwent gadoxetic acid-enhanced MRI and hepatectomy. (a) The tumor showed hypointensity on the T1-weighted image. (b) The tumor was isointense to the adjacent liver parenchyma on the T2-weighted image. (c) The tumor showed hyperintensity on diffusion-weighted imaging. (d) Hypervascularity was found in the tumor in the atrial phase, but was isointense to the adjacent liver parenchyma in the venous phase of dynamic MRI study. (e) The tumor was hypointense on the Gd-EOB-DTPA-enhanced hepatobiliary phase T1-weighted image.

**Fig 3:**
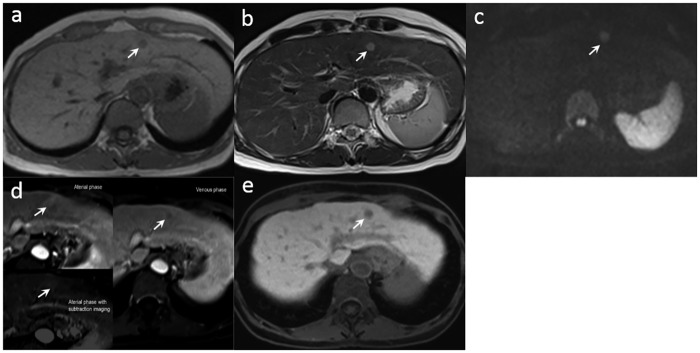
A 63-year-old male with a well-differentiated HCC (arrow) at S3 of the liver underwent gadoxetic acid-enhanced MR imaging and hepatectomy. (a) The tumor was hypointense on the T1-weighted image. (b) The tumor depicted hyperintensity on the T2-weighted image. (c) The tumor depicted hyperintensity on the diffusion-weighted image. (d)The tumor was isointense to the adjacent liver parenchyma in the arterial phase, but was hypointense in the venous phase of the dynamic MRI study. (e) The tumor was hypointense on the Gd-EOB-DTPA-enhanced hepatobiliary phase T1-weighted image.

Of the 88 atypical cirrhotic nodules found on conventional Gd-DTPA-enhanced MR images, 53 (53/88) showed hypovascular enhancement or isovascular enhancement in the arterial phase and depicted hypointense enhancement in the venous or delayed phase of the dynamic study (Fig 2). The other 35 nodules showed hypervascular enhancement in the arterial phase without contrast washout in the venous or delayed phase of the dynamic study (Fig 3).

As shown in Table 3, there were significant differences between HCC lesions and dysplastic nodules in T2W hyperintensity, T1W hypointensity, and hypervascularity in the arterial phase, typical HCC enhancement patterns on Gd-EOB-DTPA-enhanced dynamic images, or DWI hyperintensity and hypointensity on hepatocyte-phase images. The sensitivity, specificity, positive predictive value (PPV), negative predictive value (NPV), and diagnostic accuracy rates for those imaging factors are shown in Table 4. Of the 58 HCC nodules with atypical enhancement profiles on conventional Gd-DTPA MR images, 10 demonstrated typical HCC enhancement features on the following Gd-EOB-DTPA-enhanced dynamic study. Of the 48 HCCs with atypical enhancement features on both Gd-DTPA-enhanced and Gd-EOB-DTPA-enhanced images, 45 demonstrated hypointensity on images taken during the hepatocyte phase. Hypervascularity on arterial-phase dynamic MR images plus hypointensity on hepatocyte-phase images had a sensitivity of 46.6% and a specificity of 100% for diagnosing HCC. The combination of arterial hypervascular enhancement and hypointensity on hepatocyte-phase images provided better diagnostic sensitivity than atypical HCC enhancement features alone in the second dynamic MRI studies. However, three HCC nodules and five DNs showed hyperintensity/isointensity on Gd-EOB-DTPA-enhanced hepatocyte-phase images.

**Table 3:** MR characteristics of the 88 atypical cirrhotic nodules on Gd-EOB-DTPA-enhanced MR images.

		HCC (n = 58)	DN (n = 30)	*p* value
Tumor size (cm)		2.1 ± 0.9	1.8 ± 0.5	0.062
T2WI	Hyper	46	5	<0.001
	Iso/Hypo	12	25	
T1WI	Iso/Hyper	29	24	0.006
	Hypo	29	6	
Hypervascularity in arterial phase	Yes	30	5	0.001
	No	28	25	
Washout in delayed phase	Yes	12	2	0.088
	No	46	28	
Typical HCC enhancement pattern on Gd-EOB-DTPA dynamic study	Yes	10	0	0.014
	No	48	30	
Hepatocyte phase	Hypo	54	5	<0.001
	Hyper/Iso	4	25	
DWI	Hyper	48	7	<0.001
	Hypo/iso	10	23	

HCC, hepatocellular carcinoma; DN, dysplastic nodule; T2WI, T2-weighted imaging; T1WI, T1-weighted imaging; Iso, isointense; Hypo, hypointense; Hyper, hyperintense; DWI, diffusion-weighted imaging. Value was depicted as mean ± standard deviation.

**Table 4:** The diagnostic performance of MR variables in characterizing the 88 atypical cirrhotic nodules.

	Az	Sensitivity (%)	Specificity (%)	PPV (%)	NPV (%)	Accuracy (%)
Gd-DTPA-enhanced						
Hypointensity on T1	0.651	53.4	76.7	81.6	46.0	61.4
Hyperintensity on T2	0.822	81.0	83.3	90.4	69.4	81.8
Hyperintensity on AP	0.589	34.5	83.3	80.0	39.7	51.1
Hyperintensity on DWI	0.780	79.3	76.7	86.8	65.7	78.4
Gd-EOB-DTPA-enhanced						
Tumor size(threshold of 1.6cm)	0.601	74.1	43.3	71.7	46.4	63.6
Hyperintensity on T2WI	0.813	79.3	83.3	90.2	67.6	80.7
Hypointensity on T1WI	0.650	50.0	80.0	82.9	45.3	60.2
Hyperintensity on DWI	0.797	82.8	76.7	87.3	69.7	80.7
Hypervascularity on AP	0.675	51.7	83.3	85.7	47.2	62.5
Hypointensity on HB	0.882	93.1	83.3	91.5	86.2	89.8
Typical HCC enhancing pattern on Gd-EOB-DTPA dynamic study	0.586	17.2	100.0	100.0	38.5	45.5
Hypervascularity on AP plus hypointensity on HB	0.733	46.6	100.0	100.0	49.2	64.8
Hyperintensity on AP plushyper/isointensity on DP	0.675	51.7	83.3	85.7	47.7	62.5
Hypo/isointensity on AP plus hypointensity on DP	0.570	20.7	93.3	85.7	37.8	45.5

PPV, positive predict value; NPV, negative predict value, Az, Area under the ROC curve; T2WI, T2-weighted imaging; T1WI, T1- weighted imaging; DWI, diffusion-weighted imaging; AP, arterial phase; HB, hepatobiliary phase; HCC, hepatocellular carcinoma

## Discussion

Characterization of atypical cirrhotic nodules is challenging because imaging features of early HCC often overlap those of benign nodules. A previous study tested a new combination of diagnostic criteria for discrimination of early HCC from benign nodules on Gd-EOB-DTPA-enhanced MRI and found that the presence of three or more positive findings of T1 hypointensity, T2 hyperintensity, DWI hyperintensity, arterial enhancement, wash-out, HB hypointensity, and size threshold of ≥1.5 cm on Gd-EOB-DTPA-enhanced MRI, was sensitive and highly specific. [10] In this study, we used Gd-EOB-DTPA-enhanced MRI as an additional dynamic study to further characterize atypical cirrhotic nodules found on conventional Gd-DTPA-enhanced MR images. We found that only 17.2% (10/58) of the HCC lesions with atypical enhancement patterns on Gd-DTPA-enhanced MR images could be characterized correctly on MR dynamic studies with Gd-EOB-DTPA. However, when we combined hypervascularity on arterial-phase images and hypointensity on Gd-EOB-DTPA-enhanced hepatocyte-phase images as criteria for diagnosing HCC, the correct identification rate increased from 17.2% (10/58) to 46.6% (27/58) among the HCC lesions with atypical enhancement patterns on Gd-DTPA-enhanced MR images. In addition, this criterion had a specificity of 100% in characterizing atypical cirrhotic nodules.

Similarly, in a recent prospective study, we used Gd-EOB-DTPA-enhanced MRI to characterize atypical cirrhotic nodules found on CT images and found that hypervascularity on arterial phase images plus hypointensity on Gd-EOB-DTPA-enhanced hepatocyte-phase images provided higher sensitivity (46.8%) than typical enhancement patterns (17.7%) and had a 100% specificity in characterizing atypical cirrhotic nodules. [11] Our findings indicate that Gd-EOB-DTPA-enhanced MR studies in combination with hepatocyte-phase imaging might provide higher diagnostic accuracy for patients in whom dynamic MR studies disclose atypical cirrhotic nodules. One possible explanation for our finding that hypervascularity on arterial-phase images combined with hypointensity on Gd-EOB-DTPA-enhanced hepatocyte-phase images resulted in an increased rate of detection of HCC is that liver parenchyma tends to exhibit strong enhancement, and furthermore, HCCs are typically hypointense on gadoxetic acid-enhanced hepatocyte-phase T1W images. [12, 13] Golfieri et al. also reported that atypical cirrhotic nodules showing arterial hypervascularity and hypointensity on hepatocyte-phase images were all malignant (100%). [14] Based on our findings, arterial hypervascularity plus hypointensity on hepatocyte-phase images can differentiate HCC from atypical cirrhotic nodules. Of the 48 (48/58) HCC nodules that showed atypical enhancement on both Gd-DTPA- and Gd-EOB-DTPA-enhanced images, 45 (45/48) showed hypointensity in the hepatocyte phase. Kudo also reported that reduced uptake in the hepatobiliary phase was suggestive of HCC or high-grade dysplasia with high malignant potential. [15] In our study, however, 5 (5/30) of the DNs showed hypointensity on hepatocyte-phase images. Studies have reported that some DNs are hypointense on gadoxetic acid-enhanced hepatocyte-phase T1W images. [16, 17] Therefore, clinicians should keep in mind that benign dysplastic nodules can appear hypointense on hepatocyte-phase images.

We found that there were significant differences between HCC lesions and dysplastic nodules on T2-weighted images and on diffusion-weighted images in patients with atypical enhancement patterns on conventional MR images. Our results agree with those reported by Kim et al. [18], who showed that most HCCs were hyperintense on T2W and diffusion-weighted images when evaluating hepatocellular carcinomas with atypical enhancement patterns on contrast-enhanced multiphasic CT studies. Studies have shown that hyperintensity on T2-weighted images is a strong risk factor at baseline for subsequent hypervascularization in hypovascular nodules in patients with chronic liver disease. [3, 19] Hyperintensity on diffusion-weighted images is known to indicate that nodular lesions may be malignant, as it probably reflects increased cellularity and vascular change. [20] According to our results, a biopsy should be carried out on cirrhotic nodules showing hyperintensity on T2-weighted or diffusion-weighted images.

In this study, we found that hypointensity on T1-weighted images can be used to differentiate between HCCs and DNs. Our finding is similar to that reported by Hussain et al., who showed that the majority of true HCC lesions were hypointense on T1-weighted images. [21] In addition, previous studies have demonstrated that larger tumor size is indicative of higher histopathologic grade. [22–24] Based on our results, we recommend that patients with atypical cirrhotic nodules showing hypointensity on T1W images should undergo fine-needle aspiration biopsy of the suspicious nodules or should be followed regularly at short-term intervals.

A recent study reported that it is difficult to accurately discriminate between small intrahepatic cholangiocarcinoma (ICCs) and HCCs using MR studies of cirrhotic livers, as the enhancement patterns of many ICCs and HCCs are often similar. Thus, small ICCs should be included in the differential diagnosis, because they also show hypointensity in the HB phase. [25]

In our study, only 10 dysplastic nodules were diagnosed with histologic examination and 20 dysplastic nodules were diagnosed based on the imaging criteria. In recent investigations [26, 27], high-grade dysplastic nodules were determined to be an important predictor of HCC development, and furthermore, were difficult to distinguish from early HCCs. HCCs and HGDNs should be considered malignant or premalignant lesions and therefore require immediate treatment, but LGDNs are considered benign nodules, which only need to be monitored periodically. Among small atypical cirrhotic nodules, hypointensity in the HB phase of Gd-EOB-DTPA-enhanced MR imaging is the most relevant diagnostic clue for differentiating low-risk nodules from high-risk nodules. [14]

There are several limitations in this study. First, the number of enrolled patients was relatively small, so further studies with larger populations are needed. Second, when patients had multiple nodules within the cirrhotic liver, only the largest three nodules were included. Further investigation of each nodule in explanted livers is necessary to characterize all cirrhotic nodules. Besides, the study was retrospective and DN was based on follow-up imaging study. Thus, benign nodules of liver could not be excluded. Third, as is common in retrospective studies, histologic data were not available for all of the atypical cirrhotic nodules included in this study. Therefore, for nodules that had not been investigated histologically, we relied on the findings reported during long-term CT or MR follow-up studies to establish a diagnosis. Finally, the highest b values in three participating institutes in this study were different, which may have influenced the detection rate of HCC. However, this limitation was unavoidable and its effect was likely negligible.

In conclusion, repeat dynamic study can improve the accuracy of diagnosing HCC in patients with atypically enhanced lesions on dynamic MR images. Hypervascularity in the arterial phase plus hypointensity on Gd-EOB-DTPA-enhanced hepatocyte-phase images can differentiate DNs from HCC in patients with atypical cirrhotic nodules.
